# Association of Glymphatic Imaging Markers With Clinical Outcomes and Cerebral Small Vessel Disease Burden After Intracerebral Hemorrhage

**DOI:** 10.1002/brb3.71856

**Published:** 2026-10-16

**Authors:** Xiao Hu, Zuoqiao Li, Zijie Wang, Yingmei Zhang, Mengqiu Zhang, Sijia Liu, Fan Chen, Jianshuo Shao, Longsheng Wang, Qi Li

**Affiliations:** ^1^ Department of Neurology The Second Affiliated Hospital of Anhui Medical University Hefei Anhui China; ^2^ Department of Neurology The First Affiliated Hospital of Chongqing Medical University Chongqing China; ^3^ Department of Radiology The Second Affiliated Hospital of Anhui Medical University Hefei Anhui China

**Keywords:** cerebral small vessel disease, free water fraction, glymphatic function, intracerebral hemorrhagem, white matter hyperintensity

## Abstract

**Objective:**

The glymphatic system plays a critical role in cerebral metabolite clearance and white matter injury. However, its clinical relevance in intracerebral hemorrhage (ICH) remains poorly understood. This study aimed to investigate imaging‐based glymphatic markers in relation to functional outcomes and cerebral small vessel disease (CSVD) burden in patients with ICH.

**Methods:**

We included 70 patients with acute supratentorial ICH who underwent diffusion tensor imaging (DTI). Glymphatic‐related imaging parameters were derived using magnetic resonance imaging (MRI), including DTI‐ALPS, free water (FW) fraction, and relative perivascular space (PVS) volume. CSVD burden was assessed using relative white matter hyperintensity (WMH) volume and number of lacunes. Functional independence was defined as a 3‐month modified Rankin Scale score of 0–2.

**Results:**

In fully adjusted logistic regression models, higher FW in deep WM was associated with a lower likelihood of functional independence at 3 months (OR = 0.59; 95% CI, 0.36–0.98; p = 0.041), with consistent findings across sequential models. Linear regression analyses further demonstrated that greater relative WMH volume was significantly associated with increased FW in deep WM (β = 0.143; 95% CI, 0.008–0.277; *p* = 0.038) and with smaller relative PVS volume in fully adjusted models. Neither FW association remained statistically significant after FDR correction. The FW–outcome association was also nonsignificant after adjustment for WMH burden, and the exploratory mediation analysis showed no statistically significant indirect effect.

**Interpretation:**

Contralateral FW showed nominal associations with functional outcome and WMH burden and may reflect diffuse CSVD‐related white matter injury. Its independent prognostic and mediating roles remain unestablished and require validation in larger longitudinal studies.

## Graphical abstract

In patients with intracerebral hemorrhage, higher free water fraction in contralateral white matter was associated with lower odds of functional independence and greater WMH burden. These associations did not remain statistically significant after FDR correction, and the FW–outcome association was nonsignificant after adjustment for WMH burden.

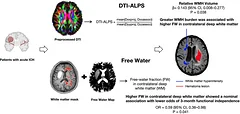

## 1 Introduction

Spontaneous intracerebral hemorrhage (ICH) is the second most common and the most lethal stroke subtype, with one‐month mortality approaching 40%, and the majority of survivors experiencing moderate to severe long‐term disability. (van Asch et al. 2010) Despite advances in acute management, substantial heterogeneity in clinical outcomes persists, underscoring the need to better understand the underlying pathophysiological mechanisms that contribute to secondary brain injury and recovery following ICH.

Cerebral small vessel disease (CSVD) represents a major public health burden and is a leading contributor to both ischemic stroke and intracerebral hemorrhage, which is also prevalent in other neurological disorders. (Markus and De Leeuw 2023) CSVD is characterized by a spectrum of lesion‐based and non‐lesion imaging markers readily detectable on magnetic resonance imaging (MRI), including white matter hyperintensities (WMH), lacunes, enlarged perivascular spaces (EPVS), cerebral microbleeds, and recent small subcortical infarcts. (Duering et al. 2023) Although CSVD is traditionally attributed to pathological changes in small cerebral arterioles, capillaries, and venules, its pathogenesis remains incompletely understood due to the complexity and interplay of vascular, inflammatory, and interstitial mechanisms. A deeper understanding of these mechanisms is essential for developing strategies to prevent irreversible brain injury associated with CSVD. (Wardlaw et al. 2019)

The glymphatic system has been proposed as a key pathway for cerebral waste clearance and interstitial fluid homeostasis, consisting of cerebrospinal fluid (CSF) flow along perivascular spaces that can be visualized using advanced MRI techniques. (Filippopulos et al. 2022) Diffusion tensor image analysis along the perivascular space (DTI‐ALPS) is a relatively non‐invasive imaging method developed to assess glymphatic function in the human brain and has been increasingly applied in studies of neurodegenerative disorders. (Taoka et al. 2017) One of the hallmark imaging features of glymphatic dysfunction is enlarged perivascular spaces, which are thought to reflect impaired clearance of cerebrospinal fluid and metabolic waste, leading to interstitial fluid accumulation. (Hong et al. 2024; Li et al. 2024) Notably, EPVS constitutes a core imaging marker of CSVD and has been proposed as a key imaging indicator of glymphatic function. Free water refers to the fraction of unconfined water molecules within brain tissue. The free water fraction (FWf), derived from diffusion tensor imaging, quantifies extracellular water that diffuses freely, distinct from tissue‐restricted diffusion influenced by cellular microstructure. This metric is considered as a sensitive indicator of microstructural alterations and has recently been proposed as a surrogate marker of glymphatic dysfunction in neurodegenerative diseases. (Li et al. 2024; Xu et al. 2025) Compared with conventional diffusion metrics, free water may provide complementary information regarding interstitial fluid dynamics and tissue integrity.

Accumulating evidence suggests that glymphatic dysfunction may influence neurological outcomes by contributing to microstructural brain injury. (Lee et al. 2024) Experimental studies in animal models have demonstrated that regional impairment of glymphatic clearance may exacerbate brain edema after acute stroke, thereby worsening neurological function. (Zhu et al. 2024; Chen et al. 2025)

However, despite cerebral small vessel disease being the most prominent underlying etiology of spontaneous intracerebral hemorrhage, few studies have systematically investigated global glymphatic function after ICH, its relationship with CSVD burden, and its potential impact on clinical outcomes in patients with ICH. This study aimed to investigate the associations between imaging‐based glymphatic markers and functional outcomes in patients with intracerebral hemorrhage, as well as their relationships with cerebral small vessel disease burden.

## 2 Materials and Methods

### 2.1 Study Population

We screened data from prospective cohorts comprising 117 adult patients with acute intracerebral hemorrhage who underwent diffusion tensor imaging (DTI) at the Second Affiliated Hospital of Anhui Medical University between January 2023 and November 2025 and at the First Affiliated Hospital of Chongqing Medical University between September 2020 and December 2021. Patients were excluded if they met any of the following criteria: (1) poor imaging quality; (2) secondary causes of intracerebral hemorrhage, including vascular malformations, tumors, or trauma; (3) infratentorial hemorrhage, primary intraventricular hemorrhage, or subarachnoid hemorrhage; or (4) loss to 3‐month follow‐up. The study protocol was approved by the Institutional Review Boards of the Second Affiliated Hospital of Anhui Medical University and the First Affiliated Hospital of Chongqing Medical University. Informed consent was obtained from all participants or their legal representatives. The study was conducted in accordance with the principles of the Declaration of Helsinki and adhered to the Strengthening the Reporting of Observational Studies in Epidemiology (STROBE) guidelines.

### 2.2 Clinical Assessment

The following data were collected for analysis: (1) demographic characteristics, including age and sex; (2) medical history and cardiovascular risk factors, including hypertension, diabetes mellitus, dyslipidemia, alcohol consumption, prior stroke, and smoking status; (3) stroke severity, assessed using the National Institutes of Health Stroke Scale (NIHSS) at baseline and during hospitalization; and (4) imaging data, acquired in DICOM format and subsequently converted to NIfTI format for further analysis. Functional outcomes at 3 months were assessed using the modified Rankin Scale (mRS) through telephone or in‐person interviews, with functional independence defined as an mRS score of 0–2.

### 2.3 Imaging Acquisition

All patients underwent at least one magnetic resonance imaging (MRI) examination on a 3.0‐T scanner (MAGNETOM Verio or MAGNETOM Vida, Siemens Healthineers, at the Second Affiliated Hospital of Anhui Medical University; and MAGNETOM Skyra, Siemens Healthineers, at the First Affiliated Hospital of Chongqing Medical University). The imaging protocol included T1‐weighted three‐dimensional magnetization‐prepared rapid gradient‐echo (3D‐MPRAGE), T2*‐weighted gradient‐echo imaging, fluid‐attenuated inversion recovery (FLAIR), diffusion‐weighted imaging (DWI), diffusion tensor imaging (DTI), and susceptibility‐weighted imaging (SWI). Detailed acquisition parameters for DTI and T1 imaging are provided in Table S1.

### 2.4 MRI Processing

The schematic overview of the imaging processing pipeline is presented in Figure 1. All T1‐weighted structural images were processed using FSL (version 6.0.3; https://fsl.fmrib.ox.ac.uk/fsl). (Smith et al. 2004) Each image was first cropped to remove the neck region using the *Robustfov* tool, followed by bias‐field correction to reduce intensity inhomogeneity. Skull stripping was performed using the Brain Extraction Tool (BET) with a fractional intensity threshold of f = 0.35. The bias‐corrected brain images were then segmented into three tissue classes—gray matter (GM), white matter (WM), and cerebrospinal fluid (CSF)—using FSL's FAST algorithm. Total intracranial volume (TIV) was defined as the sum of gray matter (GM), white matter (WM), and cerebrospinal fluid (CSF) masks. White matter hyperintensities (WMH) were delineated on FLAIR images using MRICron software, and absolute WMH volume was calculated by doubling the contralateral WMH volume. Relative WMH volume was derived by normalizing the absolute WMH volume to TIV to account for inter‐individual differences in head size.

**FIGURE 1:**
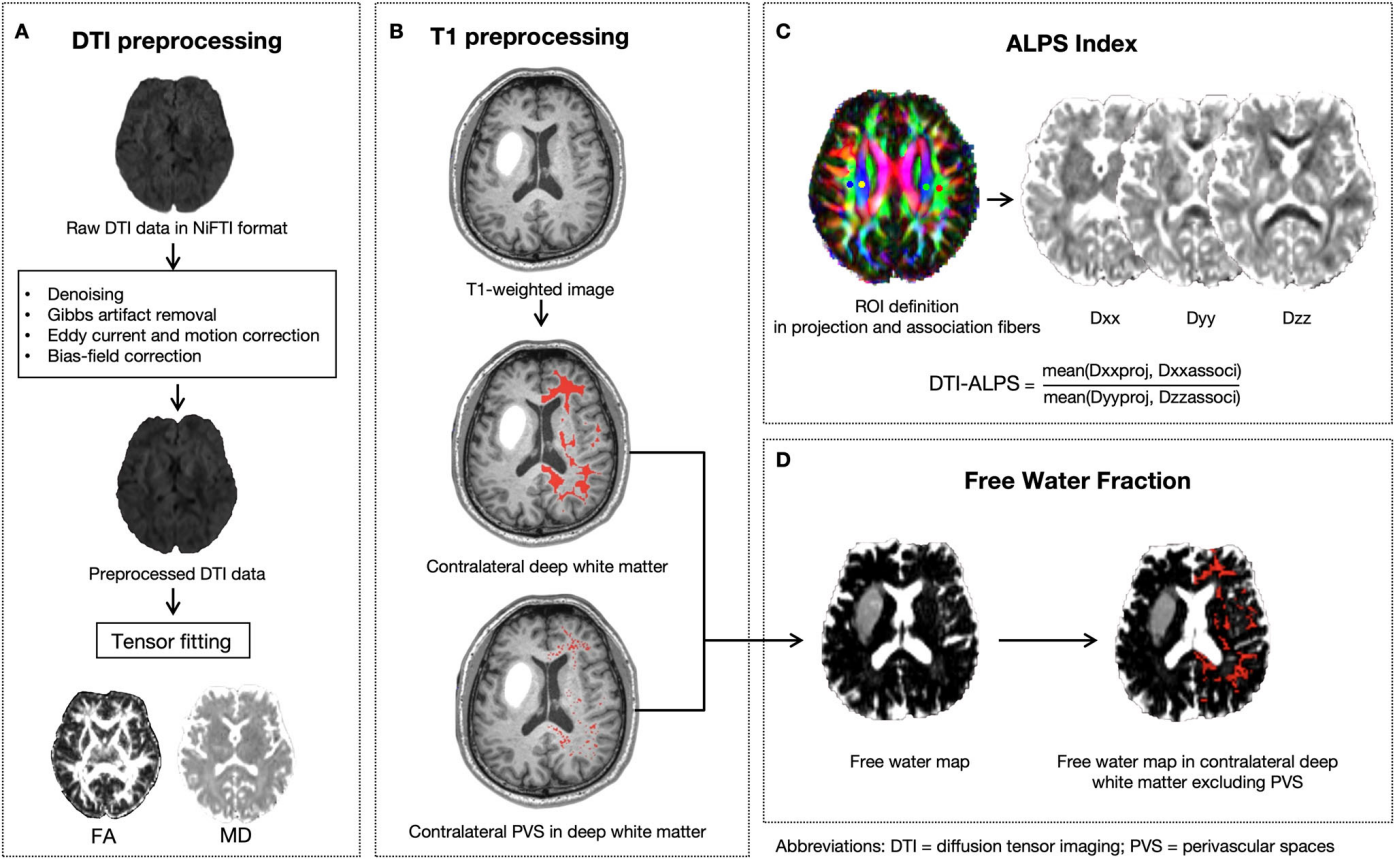
Imaging processing pipeline and derivation of imaging parameters. (A) Diffusion tensor imaging (DTI) preprocessing, including denoising, Gibbs artifact removal, eddy current and motion correction, and bias field correction, followed by tensor fitting to derive diffusion metrics. (B) Preprocessing of T1‐weighted images and generation of contralateral deep white matter and perivascular space (PVS) masks using Frangi vesselness filter. (C) Calculation of the diffusion tensor image analysis along the perivascular space (DTI‐ALPS) index based on region‐of‐interest placement within projection and association fibers. (D) Estimation of free water fraction within contralateral deep white matter after exclusion of PVS voxels.

PVS masks in the contralateral white matter were derived from T1‐weighted images following established methods. (Lin et al. 2024) Voxel‐wise PVS maps were generated by applying a multiscale Frangi vesselness filter within white matter regions, as implemented in the scikit‐image library. (Frangi et al. 1998) The Frangi filter parameters were set to α = 0.5 and β = 0.1, with a multiscale range selected to enhance tubular structures consistent with PVS morphology. The Frangi γ parameter was adaptively defined as half of the maximum Hessian norm within the white matter mask to ensure robustness across subjects. Vesselness maps were subsequently standardized using robust scaling to mitigate the influence of extreme values and thresholded at 3.4 to obtain binary PVS masks. All PVS segmentations were visually inspected after segmentation. To reduce false‐positive segmentation, PVS detection was restricted to deep white matter regions. Deep white matter masks were generated by subtracting a gray matter mask dilated by one voxel from the corresponding white matter mask. Relative PVS volume was calculated as the proportion of the contralateral deep white matter volume occupied by PVS.

DTI data were pre‐processed with a standardized pipeline that (i) suppressed thermal noise (Veraart et al. 2016), (ii) removed Gibbs‐ringing artifacts, (Kellner et al. 2016) (iii) corrected inter‐volume head motion (iv) compensated for eddy‐current–induced distortions (Andersson and Sotiropoulos 2016) (v) rectified susceptibility‐related geometric warping with topup (Andersson et al. 2003) and (vi) eliminated B_1_‐field intensity inhomogeneity. All steps were implemented with FSL (v6.0.3), MRtrix 3.0 (Tournier et al. 2019), and Advanced Normalization Tools (ANTs, v2.1.0). Brain masks were derived from the b0 volume, and diffusion tensors were subsequently computed using FSL's dtifit utility.

For calculation of DTI‐ALPS, region of interest (ROI) placement was guided by color‐coded FA maps generated from the tensor images. ROIs were manually delineated within the projection fiber regions (superior and posterior corona radiata) and association fiber regions (superior longitudinal fasciculus) using ITK‐SNAP with a 5‐mm spherical brush. Voxel‐wise diffusivities along the three principal axes (Dxx, Dyy, Dzz) were then extracted for each ROI. The DTI‐ALPS index was calculated as mean (Dxproj+Dxassoc) / mean (Dyproj+Dzassoc). (Taoka et al. 2017) Because supratentorial hematomas substantially interfere with region‐of‐interest (ROI) delineation in projection and association fibers, only the contralateral ALPS index was used in subsequent analyses.

Single‐shell free water fraction maps were computed using DIPY version 1.4.0 in Python. (Pasternak et al. 2009) For each voxel, the diffusion MRI signal was modeled using a two‐compartment approach comprising tissue and isotropic free water components, yielding a free water map that reflects the relative proportion of extracellular free water (range 0–1). The mean free water fraction in the contralateral deep white matter was calculated, with values within PVS masks further excluded to minimize the influence of fluid signal originating from PVS (Figure 1).

### 2.5 CSVD Marker Evaluation

CSVD markers on MRI were identified in accordance with the Reporting Vascular Changes on Neuroimaging (STRIVE‐2) criteria. (Duering et al. 2023) Lacunes were defined as round or ovoid cavities with signal intensity equivalent to cerebrospinal fluid on T2‐weighted and FLAIR images, measuring 3–15 mm in diameter. WMH severity was assessed on axial FLAIR images using the Fazekas scale (Fazekas et al. 1987). CMBs were defined as round or ovoid hypointense lesions on SWI. EPVS in the basal ganglia and centrum semiovale were graded using a five‐point visual scale proposed by Doubal et al. (2010).

### 2.6 Statistical Analysis

Continuous variables are presented as medians with interquartile ranges (IQRs), and categorical variables as counts and percentages. Demographic, clinical, and imaging characteristics were compared between patients with functional independence and those without using two‐sample *t*‐tests or Mann–Whitney *U* tests for continuous variables and *χ* ^2^ tests for categorical variables, as appropriate. Univariate logistic regression analyses were performed to assess the associations between clinical and imaging variables and functional independence. Linear regression models were used to examine the relationships between white matter hyperintensity (WMH) burden, total lacune count, and imaging‐derived glymphatic parameters. To assess scanner‐related variability, FW, DTI‐ALPS, and mean diffusivity measurements were harmonized using parametric empirical‐Bayes ComBat (Fortin et al. 2017), with the scanner specified as the batch variable and relevant biological and clinical covariates preserved. Residual scanner effects before and after harmonization were evaluated using HC3‐robust joint Wald (F) tests. As a sensitivity analysis, the primary outcome models were repeated using harmonized FW, DTI‐ALPS, and, where applicable, mean diffusivity values, without additional adjustment for scanner. The Benjamini–Hochberg procedure was used to control the false discovery rate across the nine fully adjusted Model 3 association tests. A two‐sided *p*‐value < 0.05 was considered statistically significant. All statistical analyses were conducted using SPSS software (version 26.0; IBM Corp., Armonk, NY, USA) and R software (version 4.1.3; R Foundation for Statistical Computing, Vienna, Austria).

## 3 Results

### 3.1 Patient Characteristics

A total of 70 patients with acute intracerebral hemorrhage were included in the study (Figure S1). Clinical and imaging characteristics were compared between patients who achieved functional independence (mRS 0–2) and those who were functionally dependent (mRS 3–6), as summarized in Table 1. The median age of the cohort was 60.0 years (IQR, 51.8‐69.0), and 32.9% of participants were female. The median interval from symptom onset to DTI acquisition was 7.0 days (IQR, 4.0‐11.0). Patients who achieved functional independence at 3 months had a lower prevalence of diabetes mellitus (18.4% vs. 42.9%, *p* = 0.032) and a lower baseline NIHSS score (median 4.0 vs. 11.0, *p* < 0.001). In contrast, patients in the dependent group exhibited a greater burden of WMH, as reflected by higher Fazekas scores, larger absolute and relative WMH volumes, and a higher lacune count (median 2.0 vs. 0.0, *p* = 0.004). Additionally, the functionally independent group showed a lower free water fraction in contralateral deep white matter (2.76×10^−^^2^ vs. 4.02 × 10^−^^2^, *p* < 0.001), while a higher relative PVS volume was also observed (19.17% vs. 17.27%, *p* < 0.001).

**TABLE 1:** Clinical and imaging characteristics.

	Total (*n* = 70)	mRS 0–2 (*n* = 49)	mRS 3–6 (*n* = 21)	*p*‐value
**Clinical characteristics**				
Age, years	60.0 (51.8‐69.0)	58.0 (48.5‐67.0)	58.0 (58.0‐73.0)	0.106
Sex, female	23 (32.9)	15 (30.6)	8 (38.1)	0.541
Hypertension	68 (97.1)	48 (98.0)	20 (95.2)	0.513
Diabetes mellitus	18 (25.7)	9 (18.4)	9 (42.9)	0.032
Current smoking	19 (27.1)	15 (30.6)	4 (19.0)	0.319
Alcohol overuse	14 (20.0)	10 (20.4)	4 (19.0)	1.000
Baseline NIHSS	4.5 (2.0‐11.0)	4.0 (2.0‐7.5)	11.0 (7.5‐18.0)	<0.001
**Imaging characteristics**				
CSO‐EPVS ≥20	20 (28.6)	15 (30.6)	5 (23.8)	0.564
BG‐EPVS ≥20	7 (10.0)	2 (4.1)	5 (23.8)	0.037
Presence of lacune	35 (50.0)	19 (38.8)	16 (76.2)	0.004
Total lacune count	0.5 (0.0‐3.0)	0.0 (0.0‐2.0)	2.0 (0.5‐4.5)	0.004
Presence of CMB	59 (84.3)	40 (81.6)	19 (90.5)	0.292
Total CMB count	5.0 (1.0‐15.5)	3.0 (1.0‐12.0)	9.5 (2.3‐27.8)	0.032
Fazekas score	3.0 (2.0‐5.0)	2.0 (2.0‐5.0)	5.0 (2.5‐6.0)	0.009
Relative WMH volume, %	0.74 (0.35‐1.78)	0.61 (0.26‐1.47)	1.22 (0.57‐2.30)	0.019
Time from onset to DTI, days	7.0 (4.0‐11.0)	7.0 (4.0‐10.0)	7.0 (3.0‐14.0)	0.690
Scanner				
MAGNETOM Vida	44 (62.9)	31 (63.3)	13 (61.9)	0.040
MAGNETOM Verio	13 (18.6)	6 (12.2)	7 (33.3)	
MAGNETOM Skyra	13 (18.6)	12 (24.5)	1 (4.8)	
MD in Contralateral deep WM, 10^−3^	0.823 (0.796‐0.848)	0.814 (0.793‐0.837)	0.843 (0.816‐0.884)	0.003
Baseline hematoma volume	5.77 (3.06‐10.01)	5.93 (3.02‐9.84)	5.60 (3.30‐12.13)	0.873
Relative PVS volume, %	18.75 (17.04‐19.91)	19.17 (18.12‐20.28)	17.27 (15.61‐18.57)	<0.001
Contralateral DTI‐ALPS index	1.350 (1.215‐1.504)	1.356 (1.242‐1.540)	1.338 (1.100‐1.450)	0.172
FW in contralateral deep WM, 10^−2^	3.04 (2.45‐3.97)	2.76 (2.32‐3.44)	4.02 (3.20‐5.68)	<0.001
FW in contralateral deep WM excluding PVS, 10^−2^	3.08 (2.55‐4.18)	2.90 (2.43‐3.52)	4.18 (3.31‐5.86)	<0.001

Data are presented as median (interquartile range) or n (%), as appropriate.

Abbreviations: BG‐EPVS = enlarged perivascular spaces in basal ganglia; CMB = cerebral microbleeds; CSO‐EPVS = enlarged perivascular spaces in centrum semiovale; CSVD = cerebral small vessel disease; DTI‐ALPS = diffusion tensor image analysis along the perivascular space; FW = free water; ICH = intracerebral hemorrhage; MD = mean diffusivity; mRS = modified Rankin Scale; NIHSS = National Institutes of Health Stroke Scale; PVS = Perivascular spaces; WMH = White matter hyperintensities.

### 3.2 Association Between Glymphatic Parameters and Functional Independence

In univariate logistic regression analyses, demographic factors, including age (OR = 0.92, 95% CI 0.88–0.97; *p* = 0.003) and diabetes mellitus (OR = 0.30, 95% CI 0.09–0.98; *p* = 0.045), as well as imaging characteristics, including lacune presence, total lacune count, relative WMH volume, and the contralateral free water, both excluding PVS (OR = 0.49, 95% CI 0.29–0.82; *p* = 0.007) and including PVS (OR = 0.51, 95% CI 0.30–0.87; *p* = 0.013), were associated with functional independence (Table S2).

In Model 2, free water in deep white matter excluding PVS remained significantly associated with functional independence after adjustment for age, hematoma volume, and diabetes mellitus (OR = 0.54, 95% CI 0.30–0.97; *p* = 0.040). This association persisted after further adjustment for scanner effects in Model 3 (OR = 0.59, 95% CI 0.36–0.98; *p* = 0.041; Table 2) and was modestly attenuated when the free water in deep white matter, including PVS, was used (OR = 0.62, 95% CI 0.38–1.00; *p* = 0.052; Table S3, Model 3). After additional adjustment for onset‐to‐DTI interval, the association between contralateral FW and 3‐month functional independence remained similar (OR per 0.01 increase, 0.57; 95% CI, 0.33–0.99; *p* = 0.047). The timing covariate was not independently associated with functional independence (OR per day, 1.02; 95% CI, 0.89–1.18; *p* = 0.768, Table S4).

**TABLE 2:** Association between glymphatic imaging parameters and functional independence.

	OR (95% CI)	*p* value		OR (95% CI)	*p*‐value		OR (95% CI)	*p*‐value
**Model 1**			**Model 1**			**Model 1**		
FW in deep WM^a^	0.49 (0.29 to 0.82)	0.007	Contralateral ALPS^b^	1.02 (0.99 to 1.05)	0.161	Relative PVS volume^c^	1.32 (0.85 to 2.05)	0.220
**Model 2**			**Model 2**			**Model 2**		
FW in deep WM^a^	0.54 (0.30 to 0.97)	0.040	Contralateral ALPS^b^	1.01 (0.98 to 1.04)	0.649	Relative PVS volume^c^	1.17 (0.70 to 1.94)	0.562
Age	0.96 (0.90 to 1.01)	0.129	Age	0.94 (0.89 to 0.99)	0.020	Age	0.95 (0.89 to 1.01)	0.096
Hematoma volume	0.99 (0.92 to 1.08)	0.893	Hematoma volume	0.99 (0.92 to 1.08)	0.882	Hematoma volume	0.99 (0.91 to 1.07)	0.734
Diabetes	0.37 (0.10 to 1.35)	0.133	Diabetes	0.39 (0.11 to 1.37)	0.142	Diabetes	0.45 (0.13 to 1.59)	0.214
**Model 3**			**Model 3**			**Model 3**		
FW in deep WM^a^	0.59 (0.36 to 0.98)	0.041	Contralateral ALPS^b^	1.00 (0.97 to 1.03)	0.886	Relative PVS volume^c^	1.21 (0.95 to 1.55)	0.125
Age	0.95 (0.89 to 1.02)	0.156	Age	0.94 (0.88 to 1.00)	0.068	Age	0.95 (0.89 to 1.02)	0.181
Hematoma volume	0.99 (0.90 to 1.09)	0.821	Hematoma volume	0.98 (0.90 to 1.07)	0.663	Hematoma volume	0.97 (0.89 to 1.07)	0.562
Diabetes	0.32 (0.08 to 1.27)	0.105	Diabetes	0.32 (0.09 to 1.17)	0.085	Diabetes	0.38 (0.10 to 1.44)	0.152
Scanner			Scanner			Scanner		
Vida	reference		Vida	reference		Vida	reference	
Verio	1.11 (0.22 to 5.59)	0.902	Verio	0.65 (0.15 to 2.89)	0.574	Verio	0.78 (0.17 to 3.54)	0.745
Skyra	4.41 (0.41 to 47.84)	0.223	Skyra	6.92 (0.70 to 68.17)	0.097	Skyra	14.50 (0.71 to 297.91)	0.083

Abbreviations: ALPS = Diffusion tensor image‐analysis along the perivascular space; CI = confidence interval; FW = free water; mRS = modified Rankin Scale; OR = Odds ratio; PVS = perivascular spaces; WM = white matter.

Notes: Functional independence was defined as a 3‐month mRS score of 0–2.

^a^ FW in deep WM was calculated as the mean FW fraction within contralateral deep WM after exclusion of PVS and was multiplied by 100.

^b^ The DTI‐ALPS index was derived from the contralateral hemisphere and multiplied by 100 to improve interpretability of effect estimates.

^c^ Relative PVS volume was calculated as the proportion of PVS volume within contralateral deep white matter, normalized to contralateral deep white matter volume.

ComBat harmonization reduced the residual scanner effect for contralateral FW from (*p* = 0.002) before harmonization to (*p* = 0.856) afterward. No significant scanner effect was detected for DTI‐ALPS either before or after harmonization (*p* = 0.672 and *p* = 0.816, respectively). Higher harmonized FW remained significantly associated with lower odds of functional independence (OR per 0.01‐unit increase, 0.54; 95% CI, 0.33–0.89; *p* = 0.016), consistent with the original scanner‐adjusted analysis; DTI‐ALPS remained nonsignificant (Table S5). However, the FW‐outcome association did not remain statistically significant after FDR correction (FDR‐corrected *p* = 0.092; Table S6).

Of the 70 included patients, 61 (87.1%) had deep ICH and 9 (12.9%) had lobar ICH. In an exploratory analysis restricted to patients with deep ICH, higher contralateral FW remained directionally associated with lower odds of functional independence, although the association was not statistically significant after full adjustment (OR per 0.01‐unit increase, 0.66; 95% CI, 0.40–1.08; *p* = 0.096; Table S7). In contrast, no significant associations were observed between the contralateral DTI‐ALPS index or relative perivascular space volume and functional independence across any of the models (Table 2).

In a sensitivity analysis additionally adjusting for log‐transformed relative WMH volume, the association between contralateral FW and 3‐month functional independence was modestly attenuated and no longer statistically significant (OR 0.62, 95% CI 0.36–1.06; *p* = 0.080; Table S8). The exploratory mediation analysis also showed no statistically significant indirect effect of WMH burden on functional independence through contralateral FW (ACME −0.036, 95% CI −0.106 to 0.005; *p* = 0.101; Table S9). No significant interactions were observed between the free water in contralateral deep white matter excluding PVS and either WMH burden or total lacune count with respect to functional independence (all interaction *p*‐values > 0.05, Table S10).

### 3.3 Associations of Relative White Matter Hyperintensity Volume and Total Lacune Count with Imaging‐Based Glymphatic Markers

Linear regression analyses were conducted to examine the associations between glymphatic parameters and relative WMH volume across different models (Table 3). A higher free water in contralateral deep white matter excluding PVS was consistently and significantly associated with greater relative WMH volume in all three models after adjustment for age, NIHSS score, baseline hematoma volume, mean diffusivity of contralateral deep white matter, and scanner effects (β = 0.143; 95% CI 0.008–0.277; *p* = 0.038). In contrast, a negative association between relative WMH volume and relative PVS volume was observed across all models (*p* < 0.001). With respect to total lacune count, the free water in contralateral deep white matter excluding PVS showed a positive association in the unadjusted model (β = 0.130; 95% CI 0.034–0.226; *p* = 0.008) and remained significant in Model 2 after adjustment for age, admission NIHSS score, and baseline hematoma volume (β = 0.138; 95% CI 0.053–0.222; *p* = 0.002, Table 4). However, this association was attenuated and no longer statistically significant after further adjustment for mean diffusivity and scanner effects. In contrast, relative PVS volume was inversely associated with total lacune count in the fully adjusted model (β = −0.201; 95% CI −0.342 to −0.060; *p* = 0.006), and this inverse association was consistently observed in the other two models as well (Table 4). In fully adjusted models, WMH burden was not significantly associated with BG‐EPVS grade (common OR, 1.53; 95% CI, 0.89–2.62; FDR‐corrected *p* = 0.243) or CSO‐EPVS grade (common OR, 0.99; 95% CI, 0.64–1.55; FDR‐corrected *p* = 0.975; Table S11). Restricted cubic spline analyses demonstrated no evidence of a non‐linear association between relative WMH volume or total lacune count and glymphatic imaging parameters (Figure S2).

**TABLE 3:** Linear regression analyses between relative WMH volume and glymphatic parameters.

	FW in deep WM^a^		Contralateral ALPS^b^		Relative PVS volume^c^	
	β coefficient (95% CI)	*p*‐value	β coefficient (95% CI)	*p*‐value	β coefficient (95% CI)	*p*‐value
Model 1	0.692 (0.361 to 1.024)	< 0.001	−3.639 (−9.360 to 2.081)	0.209	−1.222 (−1.552 to −0.893)	< 0.001
Model 2	0.578 (0.258 to 0.899)	0.001	−1.132 (−8.022 to 5.758)	0.744	−0.974 (−1.412 to −0.536)	< 0.001
Model 3	0.143 (0.008 to 0.277)	0.038	2.846 (−3.655 to 9.346)	0.385	−0.858 (−1.234 to −0.481)	< 0.001

Model 1: Unadjusted.

Model 2: Adjusted for age, admission NIHSS score, and baseline hematoma volume.

Model 3: Adjusted for age, admission NIHSS score, baseline hematoma volume, mean diffusivity of contralateral deep white matter, and scanner.

*Note*: Relative WMH volume was calculated as absolute WMH volume×2 divided by intracranial volume, and subsequently log‐transformed.

^a^ FW in deep WM was calculated as the mean FW fraction within contralateral deep WM after exclusion of PVS and was multiplied by 100.

^b^ The DTI‐ALPS index was derived from the contralateral hemisphere and multiplied by 100 to improve interpretability of effect estimates.

^c^ Relative PVS volume was calculated as the proportion of PVS volume within contralateral deep white matter, normalized to contralateral deep white matter volume.

Abbreviations: ALPS = diffusion tensor image‐analysis along the perivascular space; CI = confidence interval; FW = free water; NIHSS = National Institutes of Health Stroke Scale; PVS = perivascular spaces; WM = white matter.

**TABLE 4:** Linear regression analyses between total lacune Count and glymphatic parameters.

	FW in deep WM^a^		Contralateral ALPS^b^		Relative PVS volume^c^	
	β coefficient (95% CI)	*p* value	β coefficient (95% CI)	*p* value	β coefficient (95% CI)	*p* value
Model 1	0.130 (0.034 to 0.226)	0.008	0.451 (−1.365 to 2.266)	0.622	−0.245 (−0.484 to −0.007)	0.044
Model 2	0.138 (0.053 to 0.222)	0.002	0.303 (−1.623 to 2.228)	0.755	−0.255 (−0.448 to −0.062)	0.010
Model 3	0.024 (−0.069 to 0.116)	0.610	1.227 (−0.421 to 2.876)	0.142	−0.201 (−0.342 to −0.060)	0.006

Model 1: Unadjusted.

Model 2: Adjusted for age, admission NIHSS score, and baseline hematoma volume.

Model 3: Adjusted for age, admission NIHSS score, baseline hematoma volume, mean diffusivity of contralateral deep white matter, and scanner.

^a^ FW in deep WM was calculated as the mean FW fraction within contralateral deep WM after exclusion of PVS and was multiplied by 100.

^b^ The DTI‐ALPS index was derived from the contralateral hemisphere and multiplied by 100 to improve interpretability of effect estimates.

^c^ Relative PVS volume was calculated as the proportion of PVS volume within contralateral deep white matter, normalized to contralateral deep white matter volume.

Abbreviations: CI = confidence interval; DTI‐ALPS = Diffusion tensor image‐analysis along the perivascular space; FW = free water; NIHSS = National Institutes of Health Stroke Scale; PVS = perivascular space; WM = white matter.

## 4 Discussion

In this study, we found that lower free water in contralateral deep white matter, excluding perivascular spaces, was associated with a higher likelihood of functional independence in patients with intracerebral hemorrhage. Higher free water was associated with greater relative WMH volume across all models. These findings suggest that contralateral FW may reflect diffuse white matter injury and may have potential relevance to functional outcomes after intracerebral hemorrhage.

FW fraction is derived from a two‐compartment diffusion model and estimates the isotropic extracellular/free‐water signal within each voxel. (Pasternak et al. 2009) Increases in FW can be detected independently of changes in FA or MD and have been associated with the progression of neurodegenerative diseases. These findings suggest that FW captures extracellular pathological alterations rather than serving as a nonspecific diffusion measure. (Planetta et al. 2016) Given that the glymphatic system regulates cerebrospinal fluid‐interstitial fluid exchange and interstitial fluid clearance, alterations in extracellular water content within white matter have been proposed as an indirect imaging correlate of glymphatic dysfunction. (Li et al. 2024) Accordingly, elevated FW may indicate impaired interstitial fluid homeostasis and clearance and may also capture related processes such as blood‐brain barrier disruption, neuroinflammation, or vasogenic edema. (Ray et al. 2023) Therefore, free water should be regarded as a fluid‐sensitive microstructural marker that is relevant to glymphatic function, rather than a direct measure of glymphatic flow. Previous evidence has shown that impaired glymphatic function, as reflected by ipsilateral DTI‐ALPS indices, can independently predict long‐term outcomes in patients with intracerebral hemorrhage. (Xia et al. 2024) Moreover, experimental studies have demonstrated that upregulation of aquaporin‐4 enhances glymphatic clearance capacity and is associated with reduced hematoma burden and improved neurological outcomes in animal models, suggesting that glymphatic function may contribute to the clearance of hematoma‐derived byproducts and thereby modulate secondary injury processes and clinical outcomes following intracerebral hemorrhage. (Chen et al. 2025) Consistent with the present findings, each 0.01 increase in FW was associated with a 41% lower odds of functional independence in the fully adjusted model. The FW‐outcome association remained consistent after ComBat harmonization, with no residual scanner effects detected. This reduces the likelihood that the association was driven solely by scanner variability, although residual hardware‐ or protocol‐related bias cannot be excluded given the small, uneven scanner‐specific samples. To avoid overestimation of the FW fraction and to better capture global glymphatic‐related alterations rather than effects driven by perilesional pathology, PVS were explicitly excluded from the FW calculation. Accordingly, our results suggest that alterations in glymphatic‐related imaging markers may be associated with functional outcomes by impairing metabolite clearance and disrupting the regulation of interstitial fluid influx and efflux.

The association between glymphatic system dysfunction and CSVD has been recognized in previous studies. In the present study, a greater burden of white matter hyperintensities was significantly associated with a higher FW fraction in the contralateral deep white matter, a region unaffected by the ipsilateral hematoma and explicitly excluding perivascular spaces. The positive association between WMH burden and contralateral FW suggests that FW may partly reflect global CSVD‐related white matter microstructural damage. Although adjustment for WMH burden resulted in only modest attenuation of the estimated association between contralateral FW and functional independence, the corresponding confidence interval included unity (Table S8). However, as the associations involving contralateral FW did not remain statistically significant after FDR correction, they should be regarded as suggestive and require confirmation in larger cohorts. The direction of the estimated indirect effect warrants further investigation in adequately powered longitudinal studies. Another CSVD imaging marker, total lacune count, also showed a positive association with FW; however, this relationship was attenuated after further adjustment for mean diffusivity and scanner‐related effects, indicating partial dependence on underlying microstructural integrity. Prior population‐based studies have proposed FW as an intermediate imaging phenotype linking perivascular diffusion abnormalities to structural white matter damage and executive dysfunction in aging populations. (Liu et al. 2025; Won et al. 2025) CSVD is characterized by chronic endothelial dysfunction, blood‐brain barrier disruption, and impaired perivascular drainage, all of which may lead to inefficient interstitial fluid clearance. (Wardlaw et al. 2019) Because FW reflects extracellular and interstitial fluid accumulation at the voxel level, structural alterations of small vessels and perivascular spaces may disrupt cerebrospinal fluid‐interstitial fluid exchange and clearance, resulting in extracellular fluid retention detectable as increased FW and reduced perivascular diffusivity. (Lin et al. 2024) Notably, although lacunes exhibited associations with glymphatic‐related parameters that were directionally consistent but weaker in magnitude, other cerebral small vessel disease imaging markers showed little or no association with functional independence. These findings underscore the mechanistic heterogeneity of CSVD, with different imaging markers reflecting distinct underlying pathological processes and demonstrating differential relationships with glymphatic‐related parameters. (Brenlla et al. 2025; Tian et al. 2022; Lee et al. 2024) Together, these findings suggest that different CSVD imaging features reflect distinct underlying pathophysiological processes and therefore exhibit differential relationships with glymphatic‐relevant imaging markers. Within this framework, FW may be interpreted as a fluid‐sensitive microstructural marker reflecting impaired interstitial homeostasis, rather than as a direct upstream contributor to CSVD pathology.

In the present study, no significant association was observed between the DTI‐ALPS index and either clinical outcomes or CSVD burden, as assessed by white matter hyperintensity volume and total lacune count. Although the DTI‐ALPS index is widely used as a surrogate marker of perivascular fluid transport, it is susceptible to scanner‐related variability and ROI placement. In contrast, free water may function as an intermediate imaging marker linking perivascular dysfunction to white matter damage, capturing early CSVD‐related microstructural alterations. (Zhang et al. 2025) Notably, recent evidence from postmortem brain studies has demonstrated that the ALPS index is partly driven by microstructural asymmetry of white matter fibers rather than purely reflecting perivascular fluid transport, indicating that ALPS is influenced by intrinsic diffusion tensor geometry rather than glymphatic flow alone. (Li et al. 2025) Taken together, these findings support our observation that the free water fraction may represent a more sensitive imaging marker of white matter injury in the context of CSVD.

In addition to free water and DTI‐ALPS, relative PVS volume in the contralateral deep white matter showed a counterintuitive negative association with both WMH volume and total lacune count. Although enlarged PVS are commonly regarded as an imaging marker of small vessel disease and impaired cerebrospinal fluid clearance, this inverse relationship is likely attributable to both methodological and pathophysiological factors. In the present study, PVS volume was derived using Frangi filter‐based segmentation to suppress vascular signals, thereby capturing the structural component of the perivascular drainage network. (Lin et al. 2024) However, severe white matter damage may hinder reliable PVS identification, as WMH and lacunes can obscure typical PVS morphology through gliosis, tissue rarefaction, or cavitation, leading to underestimation of measurable PVS volume in individuals with greater CSVD burden. (Duering et al. 2023) Conventional visual EPVS grading did not reproduce the inverse association between automated relative PVS volume and WMH burden. Consequently, the inverse association may partly reflect measurement characteristics rather than a direct biological reduction in PVS burden. Therefore, this automated finding should be considered method‐dependent and exploratory rather than definitive evidence that advanced CSVD reduces PVS burden. Therefore, the observed inverse association likely reflects impaired detectability and structural degradation of perivascular spaces in advanced disease, rather than a true protective effect.

This study has several limitations. First, only patients with supratentorial intracerebral hemorrhage who were able to undergo diffusion tensor imaging were included; consequently, patients with more severe strokes or larger hematoma volumes may have been underrepresented, introducing potential selection bias. Second, the analysis primarily focused on contralateral imaging parameters as surrogate markers of global glymphatic function, whereas perilesional glymphatic alterations were not specifically evaluated. Third, automated relative PVS volume showed limited concordance with visually rated EPVS. Confluent WMH and structural tissue damage may have reduced PVS conspicuity and affected automated segmentation. Accordingly, the inverse association between relative PVS volume and WMH burden should be considered method‐dependent and exploratory until the automated measure has been further validated against established visual or manual assessment methods. Fourth, the modest sample size limited the precision of the estimates, the number of covariates that could be included in the multivariable models, and the statistical power of analyses accounting for multiple comparisons. Associations involving FW did not remain statistically significant after FDR correction and should therefore be interpreted as suggestive. In addition, only nine patients had lobar ICH, precluding reliable subgroup or interaction analyses of differences according to hemorrhage location or underlying small‐vessel etiology. These findings should be validated in larger, independent cohorts.

## 5 Conclusion

In conclusion, this study demonstrates that the free water fraction in contralateral, unaffected white matter is inversely associated with functional independence and positively associated with WMH burden in patients with intracerebral hemorrhage. These findings suggest that contralateral free water may represent a sensitive imaging marker of diffuse chronic white matter injury relevant to functional outcomes after ICH, supporting further evaluation of FW as a potential imaging marker in larger independent cohorts.

## Author Contributions

X. H. and Q. L. conceptualized the study. X. H. and Z. W. developed the methodology. **Xiao Hu** performed the formal analysis. X. H., Z. L., Z. W., Y. Z., M. Z., S. L., F. C., and J. S. contributed to data curation and investigation. Q. L. supervised the study. L. W. and Q. L. acquired funding. X. H., Z. L., and Z. W. drafted the manuscript. All authors reviewed the manuscript and approved the final version.

## Funding

This study was supported by the National Natural Science Foundation of China (No. 82471368), the Noncommunicable Chronic Disease‐National Science and Technology Major Project (No. 2025ZD0552602), the Excellent Research and Innovation Team Project of Anhui Province (No. 2024AH010014), the Health Research Program of Anhui (No. 2024Aa40015), and the Clinical and Translational Research Project of Anhui Province (No. 202427b10020090 and No. 202427b10020083).

## Ethics Statement

The study protocol was approved by the Institutional Review Boards of the Second Affiliated Hospital of Anhui Medical University (No. YX2024‐068 and No. YX2025‐174) and the First Affiliated Hospital of Chongqing Medical University (No. 2025‐413‐01).

## Consent

Informed consent was obtained from all participants or their legal representatives.

## Conflicts of Interest

The authors declare no conflicts of interest.

## Supporting information




**Supplementary Material**: brb371856‐sup‐0001‐SuppMat.docx

## Data Availability

The de‐identified data supporting this study's findings will be made available upon reasonable request by the corresponding author.
