# Supplementary material for: Association of Glymphatic Imaging Markers With Clinical Outcomes and Cerebral Small Vessel Disease Burden After Intracerebral Hemorrhage
Source: Brain Behav. 2026 Oct 16;16(10):e71856. doi: 10.1002/brb3.71856 (PMC13674732; doi:10.1002/brb3.71856)
Supplement: Supplementary file 1 [file BRB3-16-e71856-s001.docx]

**Supplementary Material**

**Association of Glymphatic Imaging Markers with Clinical Outcomes and Cerebral Small Vessel Disease Burden After Intracerebral Hemorrhage**

**Table of contents**

**Table S1. MRI Acquisition Parameters**

**Table S2. Univariate Logistic Regression Analyses of Clinical and Imaging Variables Associated with Functional Independence**

**Table S3. Association Between Free Water Fraction in Contralateral Deep White Matter including PVS and Functional Independence**

**Table S4. Sensitivity Analysis of the Association Between Contralateral Free-Water Fraction and 3-Month Functional Independence After Adjustment for the Onset-to-DTI Interval**

**Table S5. Residual Scanner Effects and Associations With 3-Month Functional Independence Before and After ComBat Harmonization**

**Table S6. FDR-Adjusted Results From the Fully Adjusted Association Analyses**

**Table S7. Sensitivity Analyses of the Associations Between Glymphatic Imaging Markers and 3-Month Functional Independence in Patients With Deep Intracerebral Hemorrhage**

**Table S8. Sensitivity Analysis of the Association Between Contralateral Free-Water Fraction and 3-Month Functional Independence After Additional Adjustment for WMH Burden**

**Table S9. Exploratory Mediation Analysis of the Association Between Relative WMH Volume and 3-Month Functional Independence Through Contralateral Free-Water Fraction**

**Table S10. Interaction Analyses of Relative White Matter Hyperintensity Volume and Total Lacune Count With Free-Water Fraction in Relation to 3-Month Functional Independence**

**Table S11. Associations Between Relative White Matter Hyperintensity Burden and Visually Rated Enlarged Perivascular Spaces**

**Figure S1. Flowchart of Patient Selection.**

**Figure S2. Restricted Cubic Spline Analyses of the Associations Between Glymphatic Imaging Parameters and Relative White Matter Hyperintensity Volume and Total Lacune Count.**

**STROBE checklist**

**Table S1. MRI Acquisition Parameters**

| **Center** | **Sequence** | **Scanner** | **Strength (T)** | **TR (s)** | **TE (s)** | **Flip Angle (°)** | **Slice Thickness (mm)** | **b Value (s/mm²)** | **No. of Directions** |
| --- | --- | --- | --- | --- | --- | --- | --- | --- | --- |
| **CQMU** | 3D-T1WI | Siemens Skyra | 3 | 2 | 0.0248 | 8 | 1 |  |  |
| **CQMU** | DTI | Siemens Skyra | 3 | 3.4 | 0.073 | 90 | 3 | 1000 | 30 |
| **AHMU** | 3D-T1WI | Siemens Vida | 3 | 2.3/1.9/1.88 | 0.00298/0.00421 | 9 | 1.1/1 |  |  |
| **AHMU** | DTI | Siemens Vida | 3 | 8.4/4.6/5.1/6.0 | 0.084/0.075 | 90 | 3/4/2 | 1000 | 24/30/64 |
| **AHMU** | 3D-T1WI | Siemens Verio | 3 | 1.8 | 0.00298 | 9 | 1 |  |  |
| **AHMU** | DTI | Siemens Verio | 3 | 6.7 | 0.095 | 90 | 3 | 1000 | 30 |
| Abbreviations: AHMU, The Second Affiliated Hospital of Anhui Medical University; CQMU, The First Affiliated Hospital of Chongqing Medical University. | | | | | | | | | |

**Table S2. Univariate Logistic Regression Analyses of Clinical and Imaging Variables Associated With Functional Independence**

|  | OR (95% CI) | P value |
| --- | --- | --- |
| Age, years | 0.92 (0.88 to 0.97) | 0.003 |
| Sex, male | 1.40 (0.46 to 4.23) | 0.557 |
| Hypertension | 2.40 (0.01 to 629.11) | 0.758 |
| Diabetes | 0.30 (0.09 to 0.98) | 0.045 |
| Previous stroke | 0.48 (0.16 to 1.46) | 0.198 |
| Current smoker | 1.88 (0.51 to 6.93) | 0.346 |
| Alcohol overuse | 1.09 (0.28 to 4.32) | 0.903 |
| Baseline NIHSS | 0.84 (0.69 to 1.03) | 0.094 |
| Presence of lacune | 0.20 (0.06 to 0.65) | 0.008 |
| Total lacune count | 0.79 (0.65 to 0.95) | 0.015 |
| Presence of CMB | 0.23 (0.02 to 2.48) | 0.228 |
| Total CMB count | 0.97 (0.93 to 1.01) | 0.145 |
| Baseline hematoma volume | 0.98 (0.90 to 1.07) | 0.651 |
| Relative WMH volume^a^ | 0.50 (0.31 to 0.81) | 0.005 |
| Contralateral DTI-ALPS index^b^ | 1.02 (0.99 to 1.05) | 0.161 |
| Relative PVS volume | 1.32 (0.85 to 2.05) | 0.220 |
| Contralateral FW fraction in deep WM^b^ | 0.51 (0.30 to 0.87) | 0.013 |
| Contralateral FW fraction in deep WM excluding PVS^b^ | 0.49 (0.29 to 0.82) | 0.007 |
| Notes:  ^a^Relative WMH volume was log-transformed prior to analysis  ^b^Scaled as per 0.01 increase to improve interpretability of effect estimates.  Abbreviations: CMB=cerebral microbleeds; DTI-ALPS=diffusion tensor image analysis along the perivascular space; FW=free water; NIHSS=National Institutes of Health Stroke Scale; PVS=Perivascular spaces; WMH=White matter hyperintensities | | |

**Table S3. Association Between Free Water Fraction in Contralateral Deep White Matter including PVS and Functional Independence**

|  | OR (95% CI) | P value |
| --- | --- | --- |
| **Model 1** |  |  |
| FW in deep WM including PVS^a^ | 0.51 (0.30 to 0.87) | 0.013 |
| **Model 2** |  |  |
| FW in deep WM including PVS^a^ | 0.57 (0.31 to 1.03) | 0.062 |
| Age | 0.96 (0.90 to 1.01) | 0.116 |
| Hematoma volume | 1.00 (0.92 to 1.08) | 0.900 |
| Diabetes | 0.38 (0.11 to 1.37) | 0.140 |
| **Model 3** |  |  |
| FW in deep WM including PVS^a^ | 0.62 (0.38 to 1.00) | 0.052 |
| Age | 0.95 (0.89 to 1.02) | 0.144 |
| Hematoma volume | 0.99 (0.90 to 1.09) | 0.817 |
| Diabetes | 0.34 (0.08 to 1.28) | 0.108 |
| Scanner |  |  |
| Vida | reference |  |
| Verio | 1.13 (0.22 to 5.73) | 0.886 |
| Skyra | 4.71 (0.44 to 50.45) | 0.200 |
| Notes: Functional independence was defined as a 3-month mRS score of 0–2.  ^a^ FW in deep WM was calculated as the mean FW fraction within contralateral deep WM and was multiplied by 100.  Abbreviations: FW=free water; WM=white matter; PVS=perivascular spaces; mRS=modified Rankin Scale; OR=Odds ratio; CI=confidence interval. | | |

**Table S4. Sensitivity Analysis of the Association Between Contralateral Free-Water Fraction and 3-Month Functional Independence After Adjustment for the Onset-to-DTI Interval**

|  | **OR (95% CI)** | **P value** |
| --- | --- | --- |
| FW in deep WM^a^ | 0.57 (0.33–0.99) | 0.047 |
| Age | 0.95 (0.89–1.02) | 0.149 |
| Hematoma volume | 0.99 (0.90–1.09) | 0.808 |
| Diabetes | 0.30 (0.07–1.26) | 0.100 |
| Scanner |  |  |
| Vida | Reference |  |
| Verio | 1.21 (0.22–6.81) | 0.829 |
| Skyra | 4.11 (0.37–46.11) | 0.252 |
| Days from onset to DTI | 1.02 (0.89–1.18) | 0.768 |
| Notes: Functional independence was defined as a 3-month mRS score of 0–2.  ^a^ FW in deep WM was calculated as the mean FW fraction within contralateral deep WM after exclusion of PVS and was multiplied by 100.  Abbreviations: FW=free water; WM=white matter; mRS=modified Rankin Scale; OR=Odds ratio; CI=confidence interval. | | |

**Table S5. Residual Scanner Effects and Associations With 3-Month Functional Independence Before and After ComBat Harmonization**

| **Residual scanner effects** | | | | |
| --- | --- | --- | --- | --- |
| **DTI metric** | **Before ComBat, robust F** | **P value** | **After ComBat, robust F** | **P value** |
| Contralateral FW^a^ | 6.86 | 0.002 | 0.16 | 0.856 |
| Contralateral ALPS index | 0.40 | 0.672 | 0.20 | 0.816 |
| **Associations with 3-month functional independence** | | | | |
| **DTI metric** | **Original scanner-adjusted model OR (95% CI)** | **P value** | **ComBat sensitivity model**  **OR (95% CI)** | **P value** |
| Contralateral FW, per 0.01 increase | 0.59 (0.36–0.98) | 0.041 | 0.54 (0.33–0.89) | 0.016 |
| Contralateral ALPS index, per 0.01 increase | 1.00 (0.97–1.03) | 0.886 | 1.01 (0.98–1.04) | 0.524 |
| Notes: Functional independence was defined as a 3-month mRS score of 0–2.  Functional-outcome models were adjusted for age, baseline hematoma volume, and diabetes; scanner was additionally included in the original models but omitted from the ComBat models because scanner-related variation had already been addressed during harmonization.  ^a^Contralateral FW was calculated as the mean FW fraction within contralateral deep WM after exclusion of PVS and was multiplied by 100.  Abbreviations: ALPS=analysis along the perivascular space; CI=confidence interval; DTI=diffusion tensor imaging; FW=free water; OR=odds ratio. | | | | |

**Table S6. FDR-Adjusted Results From the Fully Adjusted Association Analyses**

| **Panel A. Logistic regression models for 3-month functional independence** | | | | |
| --- | --- | --- | --- | --- |
| **Outcome** | **Imaging marker** | **OR (95% CI)** | **Raw (P)** | **FDR-adjusted (P)** |
| 3-month functional independence | Contralateral FW | 0.591 (0.357–0.978) | 0.041 | 0.092 |
| 3-month functional independence | DTI-ALPS index | 1.002 (0.974–1.031) | 0.886 | 0.886 |
| 3-month functional independence | Relative PVS volume | 1.211 (0.948–1.546) | 0.125 | 0.213 |
| **Panel B. Linear regression models of glymphatic imaging markers and CSVD measures** | | | | |
| **Dependent imaging variable** | **CSVD predictor** | **β (95% CI)** |  |  |
| Contralateral FW | Log-transformed relative WMH burden | 0.143 (0.008–0.277) | 0.038 | 0.092 |
| DTI-ALPS index | Log-transformed relative WMH burden | 2.846 (−3.655 to 9.346) | 0.385 | 0.495 |
| Relative PVS volume | Log-transformed relative WMH burden | −0.858 (−1.234 to −0.481) | <0.001 | <0.001 |
| Contralateral FW | Total lacune count | 0.024 (−0.069 to 0.116) | 0.610 | 0.686 |
| DTI-ALPS index | Total lacune count | 1.227 (−0.421 to 2.876) | 0.142 | 0.213 |
| Relative PVS volume | Total lacune count | −0.201 (−0.342 to −0.060) | 0.006 | 0.027 |
| Notes: Functional independence was defined as a 3-month mRS score of 0–2.  Benjamini–Hochberg FDR correction was applied jointly across all nine fully adjusted associations shown in Panels A and B.  ^a^ Contralateral FW was calculated as the mean FW fraction within contralateral deep WM after exclusion of PVS and was multiplied by 100.  ^b^ The DTI-ALPS index was derived from the contralateral hemisphere and multiplied by 100 to improve interpretability of effect estimates.  Panel A presents logistic regression analyses with 3-month functional independence as the outcome.  Panel B presents linear regression analyses with FW, DTI-ALPS, or relative PVS volume as the dependent variable and WMH burden or lacune count as the predictor.  Abbreviations: ALPS=analysis along the perivascular space; CI=confidence interval; CSVD=cerebral small vessel disease; DTI=diffusion tensor imaging; FDR=false discovery rate; FW=free water; OR=odds ratio; PVS=perivascular spaces; WMH=white matter hyperintensity. | | | | |

**Table S7. Sensitivity Analyses of the Associations Between Glymphatic Imaging Markers and 3-Month Functional Independence in Patients With Deep Intracerebral Hemorrhage**

|  | **Model 1, OR (95% CI)** | **P value** | **Model 2, OR (95% CI)** | **P value** | **Model 3, OR (95% CI)** | **P value** |
| --- | --- | --- | --- | --- | --- | --- |
| Contralateral FW^a^ | 0.52 (0.34–0.80) | 0.003 | 0.62 (0.39–0.98) | 0.042 | 0.66 (0.40–1.08) | 0.096 |
| DTI-ALPS index^b^ | 1.02 (0.99–1.04) | 0.146 | 1.00 (0.98–1.03) | 0.797 | 1.00 (0.97–1.03) | 0.952 |
| Relative PVS volume | 1.28 (1.02–1.61) | 0.031 | 1.10 (0.86–1.41) | 0.446 | 1.15 (0.90–1.48) | 0.270 |
| Notes: Functional independence was defined as a 3-month mRS score of 0–2.  Model 1 was unadjusted. Model 2 was adjusted for age, baseline hematoma volume, and diabetes mellitus. Model 3 was additionally adjusted for scanner. These analyses were exploratory sensitivity analyses because only nine patients had lobar ICH, precluding reliable location-stratified comparisons.  ^a^ Contralateral FW was calculated as the mean FW fraction within contralateral deep WM after exclusion of PVS and was multiplied by 100.  ^b^ The DTI-ALPS index was derived from the contralateral hemisphere and multiplied by 100 to improve interpretability of effect estimates.  Abbreviations: ALPS=analysis along the perivascular space; CI=confidence interval; DTI=diffusion tensor imaging; FW=free water; ICH=intracerebral hemorrhage; OR=odds ratio; PVS=perivascular spaces. | | | | | | |

**Table S8. Sensitivity Analysis of the Association Between Contralateral Free-Water Fraction and 3-Month Functional Independence After Additional Adjustment for WMH Burden**

|  | **OR (95% CI)** | **P value** |
| --- | --- | --- |
| FW in deep WM^a^ | 0.62 (0.36–1.06) | 0.080 |
| Age | 0.95 (0.89–1.02) | 0.182 |
| Hematoma volume | 0.99 (0.90–1.09) | 0.847 |
| Diabetes | 0.32 (0.08–1.27) | 0.104 |
| Scanner |  |  |
| Vida | Reference |  |
| Verio | 1.14 (0.22–5.90) | 0.876 |
| Skyra | 4.28 (0.39–47.00) | 0.234 |
| Log-transformed relative WMH volume | 0.81 (0.40–1.65) | 0.566 |
| Notes: Functional independence was defined as a 3-month mRS score of 0–2.  ^a^ FW in deep WM was calculated as the mean FW fraction within contralateral deep WM after exclusion of PVS and was multiplied by 100.  Abbreviations: FW=free water; WM=white matter; mRS=modified Rankin Scale; OR=Odds ratio; CI=confidence interval. | | |

**Table S9. Exploratory Mediation Analysis of the Association Between Relative WMH Volume and 3-Month Functional Independence Through Contralateral Free-Water Fraction**

| **Mediation effect** | **Estimate** | **95% CI** | **P value** |
| --- | --- | --- | --- |
| ACME | −0.036 | −0.106 to 0.005 | 0.101 |
| ADE | −0.036 | −0.173 to 0.072 | 0.548 |
| Total effect | −0.072 | −0.214 to 0.030 | 0.200 |
| Proportion mediated | 0.498 | −4.368 to 5.213 | 0.274 |
| Notes: Functional independence was defined as a 3-month mRS score of 0–2.  The exposure was log-transformed relative WMH volume, and the mediator was contralateral deep white-matter FW.  Models were adjusted for age, baseline hematoma volume, diabetes mellitus, and scanner. Estimates were derived using regression-based g-computation with 5,000 bootstrap samples (n = 70). Negative estimates indicate a lower probability of functional independence. The analysis was exploratory because WMH and FW were measured contemporaneously.  Abbreviations: ACME=average causal mediation effect; ADE=average direct effect; CI=confidence interval; mRS=modified Rankin Scale; WMH=white matter hyperintensity. | | | |

**Table S10. Interaction Analyses of Relative White Matter Hyperintensity Volume and Total Lacune Count With Free-Water Fraction in Relation to 3-Month Functional Independence**

|  | **Relative WMH Volume × Free Water**  **P for interaction** | **Total Lacune Count × Free Water**  **P for interaction** |
| --- | --- | --- |
| Model 1 | 0.312 | 0.872 |
| Model 2 | 0.395 | 0.996 |
| Model 3 | 0.516 | 0.891 |
| Model 1. Unadjusted  Model 2. Adjusted for age, diabetes, baseline hematoma volume  Model 3. Adjusted for age, diabetes, baseline hematoma volume, scanner | | |

**Table S11. Associations Between Relative White Matter Hyperintensity Burden and Visually Rated Enlarged Perivascular Spaces**

| **Outcome** | **Predictor** | **Adjusted common OR**  **(95% CI)** | **Raw (P) value** | **FDR-adjusted (P) value** |
| --- | --- | --- | --- | --- |
| BG-EPVS grade | Relative WMH burden | 1.53 (0.89–2.62) | 0.122 | 0.243 |
| CSO-EPVS grade | Relative WMH burden | 0.99 (0.64–1.55) | 0.975 | 0.975 |
| Notes: CSO-PVS and BG-PVS were visually rated on a 5-point ordinal scale ranging from 0 to 4, with higher grades indicating a greater PVS burden.  Common odds ratios represent the odds of being classified into a higher EPVS grade per increase in relative WMH burden.  Models were adjusted for age, admission NIHSS score, baseline hematoma volume, mean diffusivity of the contralateral deep white matter, and scanner.  Benjamini–Hochberg FDR correction was applied across the two fully adjusted visual-rating analyses.  Abbreviations: BG=basal ganglia; CI=confidence interval; CSO=centrum semiovale; EPVS=enlarged perivascular spaces; FDR=false discovery rate; NIHSS=National Institutes of Health Stroke Scale; OR=odds ratio; WMH=white matter hyperintensity. | | | | |

**Figure S1. Flowchart of Patient Selection.** Flowchart depicting the inclusion and exclusion process of patients with intracerebral hemorrhage. Of 117 patients who underwent diffusion tensor imaging, 70 met the eligibility criteria and were included in the final analysis.

**Figure S2. Restricted Cubic Spline Analyses of the Associations Between Glymphatic Imaging Parameters and Relative White Matter Hyperintensity Volume and Total Lacune Count.** (A-C) Restricted cubic spline curves illustrating the associations of free water (FW) in contralateral white matter (WM) excluding perivascular spaces (PVS), DTI-ALPS index, and relative perivascular space (PVS) volume with relative white matter hyperintensity volume. (D-F) Restricted cubic spline curves illustrating the associations of FW in contralateral WM excluding PVS, the DTI-ALPS index, and relative PVS volume with total lacune count. Free water fraction and the DTI-ALPS index were scaled per 0.01-unit increase to improve interpretability of effect estimates. Relative white matter hyperintensity volume was calculated as absolute white matter hyperintensity volume normalized to intracranial volume and subsequently log-transformed. All models were adjusted for age, admission NIHSS score, baseline hematoma volume, mean diffusivity of contralateral deep white matter, and scanner.

STROBE Statement—Checklist of items that should be included in reports of ***cohort studies***

|  | Item No | Recommendation | Page No |
| --- | --- | --- | --- |
| **Title and abstract** | 1 | (*a*) Indicate the study’s design with a commonly used term in the title or the abstract | 1-4 |
|  |  | (*b*) Provide in the abstract an informative and balanced summary of what was done and what was found | 1-4 |
| Introduction | | | |
| Background/rationale | 2 | Explain the scientific background and rationale for the investigation being reported | 5-7 |
| Objectives | 3 | State specific objectives, including any prespecified hypotheses | 5-7 |
| Methods | | | |
| Study design | 4 | Present key elements of study design early in the paper | 8 |
| Setting | 5 | Describe the setting, locations, and relevant dates, including periods of recruitment, exposure, follow-up, and data collection | 8 |
| Participants | 6 | (*a*) Give the eligibility criteria, and the sources and methods of selection of participants. Describe methods of follow-up | 8 |
|  |  | (*b*) For matched studies, give matching criteria and number of exposed and unexposed | NA |
| Variables | 7 | Clearly define all outcomes, exposures, predictors, potential confounders, and effect modifiers. Give diagnostic criteria, if applicable | 8-14 |
| Data sources/ measurement | 8* | For each variable of interest, give sources of data and details of methods of assessment (measurement). Describe comparability of assessment methods if there is more than one group | 8-14 |
| Bias | 9 | Describe any efforts to address potential sources of bias | 8-14 |
| Study size | 10 | Explain how the study size was arrived at | NA |
| Quantitative variables | 11 | Explain how quantitative variables were handled in the analyses. If applicable, describe which groupings were chosen and why | 8-13 |
| Statistical methods | 12 | (*a*) Describe all statistical methods, including those used to control for confounding | 13-14 |
|  |  | (*b*) Describe any methods used to examine subgroups and interactions | 13-14 |
|  |  | (*c*) Explain how missing data were addressed | NA |
|  |  | (*d*) If applicable, explain how loss to follow-up was addressed | NA |
|  |  | (*e*) Describe any sensitivity analyses | NA |
| Results | | |  |
| Participants | 13* | (a) Report numbers of individuals at each stage of study—eg numbers potentially eligible, examined for eligibility, confirmed eligible, included in the study, completing follow-up, and analysed | 15 |
|  |  | (b) Give reasons for non-participation at each stage | NA |
|  |  | (c) Consider use of a flow diagram | Fig S1 |
| Descriptive data | 14* | (a) Give characteristics of study participants (eg demographic, clinical, social) and information on exposures and potential confounders | 15 |
|  |  | (b) Indicate number of participants with missing data for each variable of interest | NA |
|  |  | (c) Summarise follow-up time (eg, average and total amount) | NA |
| Outcome data | 15* | Report numbers of outcome events or summary measures over time | 15,16 |
| Main results | 16 | (*a*) Give unadjusted estimates and, if applicable, confounder-adjusted estimates and their precision (eg, 95% confidence interval). Make clear which confounders were adjusted for and why they were included | 15-19 |
|  |  | (*b*) Report category boundaries when continuous variables were categorized | NA |
|  |  | (*c*) If relevant, consider translating estimates of relative risk into absolute risk for a meaningful time period | NA |
| Other analyses | 17 | Report other analyses done—eg analyses of subgroups and interactions, and sensitivity analyses | NA |
| **Discussion** | | | |
| Key results | 18 | Summarise key results with reference to study objectives | 20 |
| Limitations | 19 | Discuss limitations of the study, taking into account sources of potential bias or imprecision. Discuss both direction and magnitude of any potential bias | 25,26 |
| Interpretation | 20 | Give a cautious overall interpretation of results considering objectives, limitations, multiplicity of analyses, results from similar studies, and other relevant evidence | 20-26 |
| Generalisability | 21 | Discuss the generalisability (external validity) of the study results | 20-26 |
| **Other information** | | | |
| Funding | 22 | Give the source of funding and the role of the funders for the present study and, if applicable, for the original study on which the present article is based | 27 |
| *Give information separately for exposed and unexposed groups.  **Note:** An Explanation and Elaboration article discusses each checklist item and gives methodological background and published examples of transparent reporting. The STROBE checklist is best used in conjunction with this article (freely available on the Web sites of PLoS Medicine at http://www.plosmedicine.org/, Annals of Internal Medicine at http://www.annals.org/, and Epidemiology at http://www.epidem.com/). Information on the STROBE Initiative is available at http://www.strobe-statement.org. | | | |
